# Economic compensation interventions to increase uptake of voluntary medical male circumcision for HIV prevention: A systematic review and meta-analysis

**DOI:** 10.1371/journal.pone.0227623

**Published:** 2020-01-15

**Authors:** Caitlin E. Kennedy, Ping Teresa Yeh, Kaitlyn Atkins, Virginia A. Fonner, Michael D. Sweat, Kevin R. O’Reilly, George W. Rutherford, Rachel Baggaley, Julia Samuelson

**Affiliations:** 1 Social and Behavioral Interventions Program, Department of International Health, Johns Hopkins Bloomberg School of Public Health, Baltimore, Maryland, United States of America; 2 Division of Global and Community Health, Department of Psychiatry and Behavioral Sciences, Medical University of South Carolina, Charleston, South Carolina, United States of America; 3 Department of Epidemiology and Biostatistics, University of California, San Francisco, San Francisco, California, United States of America; 4 Department of HIV, World Health Organization, Geneva, Switzerland; University of the Witwatersrand, SOUTH AFRICA

## Abstract

**Background:**

Economic compensation interventions may help support higher voluntary medical male circumcision (VMMC) coverage in priority sub-Saharan African countries. To inform World Health Organization guidelines, we conducted a systematic review of economic compensation interventions to increase VMMC uptake.

**Methods:**

Economic compensation interventions were defined as providing money or in-kind compensation, reimbursement for associated costs (e.g. travel, lost wages), or lottery entry. We searched five electronic databases and four scientific conferences for studies examining the impact of such interventions on VMMC uptake, HIV testing and safer-sex/risk-reduction counseling uptake within VMMC, community expectations about compensation, and potential coercion. We screened citations, extracted data, and assessed risk of bias in duplicate. We conducted random-effects meta-analysis. We also reviewed studies examining acceptability, values/preferences, costs, and feasibility.

**Results:**

Of 2484 citations identified, five randomized controlled trials (RCTs) and three non-randomized controlled trials met our eligibility criteria. Studies took place in Kenya, Malawi, South Africa, Tanzania, Uganda, Zambia, and Zimbabwe. Meta-analysis of four RCTs showed significant impact of any economic compensation on VMMC uptake (relative risk: 5.23, 95% CI: 3.13 to 8.76). RCTs of food/transport vouchers and conditional cash transfers generally showed increases in VMMC uptake, but lotteries, subsidized VMMC, and receiving a gift appeared somewhat less effective. Three non-randomized trials showed mixed impact. Six additional studies suggested economic compensation interventions were generally acceptable, valued for addressing key barriers, and motivating to men. However, some participants felt they were insufficiently motivating or necessary; one study suggested they might raise community suspicions. One study from South Africa found a program cost of US$91 per additional circumcision and US$450-$1350 per HIV infection averted.

**Conclusions:**

Economic compensation interventions, particularly transport/food vouchers, positively impacted VMMC uptake among adult men and were generally acceptable to potential clients. Carefully selected economic interventions may be a useful targeted strategy to enhance VMMC coverage.

## Introduction

Voluntary medical male circumcision (VMMC) is an effective HIV prevention intervention [1–3] and is being scaled up in 15 priority countries in East and Southern Africa. Between 2008 and 2017, over 18 million male circumcisions were performed in these countries for HIV prevention [4]. This is a great success story in the fight against HIV, and in reaching men, who have generally been less engaged than women in HIV prevention and treatment services [5]. While many countries have made great progress in expanding VMMC uptake, including achieving or nearly reaching their initial targets [4], barriers to VMMC uptake remain [6]. Current global HIV prevention targets include 25 million additional circumcisions from 2016 to 2020 [7]. While much progress has been made towards these goals, including 4.1 million men circumcised in 2018 alone [7], the target is unlikely to be reached by 2020. Additionally, while not a specific UNAIDS target, reaching men at higher risk has been noted as a priority for focused action [8]. Innovative approaches are still needed to increase uptake of VMMC among key groups in priority countries.

Several recent reviews have considered economic compensation interventions as one strategy for increasing VMMC uptake in sub-Saharan Africa [6, 9–12]. These reviews generally found that such interventions may help to increase VMMC uptake, particularly when they address specific barriers, but evidence was limited. Economic compensation, or financial incentives, have been explored for a range of HIV-related behaviors, including sexual or drug use behaviors related to HIV prevention [12, 13], HIV testing [12, 14], linkage to or engagement in HIV treatment [10, 12, 14, 15], and adherence to antiretroviral therapy [12, 15, 16]. These interventions occur within a broader social and health system context, where there may be economic incentives for other health-related behaviors (for example, prenatal care, infant vaccinations, or insecticide-treated bednets) [17]. However, many countries have concerns about integrating incentives into their health systems, worrying that they may distort health care delivery, pit interventions against each other (particularly given concerns about HIV exceptionalism), be unsustainable in the long term, or unduly induce vulnerable populations. Yet real economic barriers have been identified–such as opportunity costs for time lost from work and the cost of travel–may prevent poorer men or men from remote and rural areas from accessing desired services [6]. In this sense, economic compensation for VMMC may be a way to reduce inequity in access to this proven HIV prevention intervention.

This review seeks to examine the evidence for a set of approaches to interventions to increase VMMC uptake among adult and high-risk men: economic (financial or non-financial) compensation interventions. Our review extends previous reviews by updating the search timeline and adding complementary reviews of acceptability, values and preferences, costs, and feasibility of economic compensation interventions. Together, this evidence base provides key information needed for developing World Health Organization (WHO) guidelines on economic compensation interventions for VMMC.

## Methods

We conducted this systematic review in accordance with Preferred Reporting Items for Systematic review and Meta-Analyses (PRISMA) guidelines (S1 Fig) [18].

### Topic definition

We defined “economic compensation interventions” as interventions that provide money or gifts (in-kind compensation) in exchange for completing least some components of VMMC (e.g. they could compensate just for undergoing the first steps of VMMC education/counseling without requiring men to complete the full circumcision procedure itself) or as reimbursement for costs associated with VMMC (e.g. travel to the circumcision facility or lost wages from time off work), or that provide the opportunity to earn such compensation in the form of lotteries or other systems. Such interventions have been variously termed economic compensation interventions, financial incentives, or demand-side financial incentives [10]. We focused on potential VMMC clients, and thus excluded interventions that focused on incentives for health workers or for the health system.

### Inclusion criteria–effectiveness review

To be included in the review, a study must have: (1) used a study design with a pre/post or multi-arm comparison by economic incentive intervention exposure, (2) measured one or more of the outcomes listed below, and (3) been published as a peer-reviewed journal article or conference abstract. We followed the PICO question format to define our review criteria:

- **Population:** Uncircumcised adolescent and adult men (ages 10 or older) who are potential candidates for male circumcision for HIV prevention within public health VMMC programs
- **Intervention:** Economic compensation interventions (financial or in-kind) for accessing VMMC services
- **Comparison:** No economic compensation interventions, or a different/lesser type of intervention (such as a lower amount of compensation)
- **Outcomes:** (1) Uptake of VMMC, (2) Uptake of HIV testing within VMMC services, (3) Uptake of safer sex and risk reduction counselling within VMMC services, (4) Changes in community expectations for economic compensation for other services, (5) Potential coercion (undue influence) on individuals or groups within the community

To explore subgroup differences, we stratified outcomes by a variety of subpopulations where possible. First, we stratified by whether participants (men) were considered high-risk for HIV or not, with high-risk defined as having any one of the three following characteristics: (1) being in the three highest HIV incidence 5-year age strata per Joint United Nations Programme on HIV/AIDS (UNAIDS) country estimates, (2) having more than one sexual partner, and/or (3) having a recent history of a sexually transmitted infection (STI) other than HIV. Second, we stratified by the following age groupings: (1) adolescents age 10–14 years, (2) adolescents age 15–19 years and (3) adults age ≥20 years. Third, to incorporate a health equity lens, we stratified by PROGRESS variables: place of residence (e.g. rural/urban); race, ethnicity, culture, and language; occupation; gender/sex; religion; education; socioeconomic status; and social capital [19].

### Search strategy and screening

The search was broad, as it was developed in conjunction with another related review on service-delivery interventions to increase uptake of VMMC developed for the same WHO guidelines (manuscript under review). We searched five electronic databases (PubMed, CINAHL, Sociological Abstracts, PsycINFO, and EMBASE) and four scientific conferences (International AIDS Conference (IAC), International AIDS Society (IAS) Conference on HIV Science, Conference on Retroviruses and Opportunistic Infections (CROI), and International Conference on AIDS and STIs in Africa (ICASA)). Electronic databases were searched from January 1, 1990, through May 31, 2018. We searched the bibliographies of all studies included in the review and of several related reviews [9, 10, 20]. Further, selected experts in the field–specifically, members of the WHO Guideline Development Group for Medical Male Circumcision–were asked to suggest additional articles not identified through other search methods. We also searched for ongoing randomized controlled trials (RCTs) through clinicaltrials.gov, the WHO International Clinical Trials Registry Platform, the Pan African Clinical Trial Registry, and the Australian New Zealand Clinical Trials Registry.

The following search strategy was adapted for entry into all electronic databases: (HIV [tiab] OR “human immunodeficiency virus” [tiab]) AND (circumcision [tiab] or circumcis* [tiab] or VMMC [tiab]). Only the term “circumcision” was used to search conference abstracts because all conferences were HIV-related and search functions were limited.

Citations identified through the search were screened in duplicate. Two independent reviewers then assessed all noted full-text articles for study inclusion eligibility and resolved differences through consensus.

### Data extraction and management

Data were extracted independently and in duplicate using standardized forms. Differences in data extraction were resolved through consensus and referral to a senior study team member when necessary. The following information was gathered from each included study:

- Study identification: Author(s), type of citation, year of publication
- Study description: Study objectives, location, population characteristics, main intervention description (for all study arms), description of any additional intervention components, study design, sample size, follow-up periods, loss to follow-up
- Outcomes: Analytic approach, outcome measures, comparison groups, effect measures and sizes, confidence intervals, significance levels, conclusions, limitations

Study rigor was assessed using the Cochrane Collaboration’s tool for assessing risk of bias for RCTs [21], and the Evidence Project risk of bias tool for other study designs presenting comparative data [22].

### Data analysis

We analyzed data according to coding categories and outcomes. Where multiple studies used similar intervention approaches and reported the same outcome, we conducted meta-analysis using random-effects models to present risk ratios with the program Comprehensive Meta-Analysis (Biostat, Inc., Englewood, New Jersey, USA). Heterogeneity was assessed using both Q and I-squared statistics. Funnel plots were created to examine the potential for publication bias where there were a sufficient number of studies. Data from RCTs and non-randomized studies were meta-analysed separately.

### Additional reviews

In addition to the main effectiveness review, we conducted complementary reviews of studies examining the acceptability, values and preferences, costs, and feasibility of economic compensation interventions. These reviews added important, complementary evidence for the purpose of developing holistic WHO guidelines [23]. We used the same search terms to identify studies for these reviews. These studies could be qualitative or quantitative in nature but had to present primary data; opinion pieces, editorials, and review articles were not included. We summarized this literature qualitatively and organized it by study design and methodology, location, and population.

#### Acceptability review

The acceptability review covered studies that presented primary data examining people’s feelings about the acceptability of economic incentives. We focused on studies examining the perspectives of men who have used or potentially would use VMMC, but we also included studies examining the acceptability of the interventions among providers and other stakeholders (such as partners, families, and communities).

#### Values and preferences review

The values and preferences review covered studies that presented primary data examining people’s feelings about the five outcomes in the effectiveness review. We assessed values and preferences around population-level uptake of circumcision as an outcome, rather than feelings about circumcision itself as a procedure. We focused on studies examining the perspectives of men who have used or potentially would use VMMC, but we also included studies examining values and preferences among providers and other stakeholders (such as partners, families, communities, and policy-makers).

#### Costs review

The costs review covered studies that presented primary data examining cost or cost-effectiveness of economic incentives, or otherwise discussed resource use in relation to these interventions.

#### Feasibility review

The feasibility review covered studies that presented primary data examining issues with the delivery of economic incentives or how they fit within health systems (such as training, monitoring, evaluation, etc.). We also included studies that showed that these interventions were conducted in a given setting, an indicator of their feasibility.

## Results

### Effectiveness review

#### Search results

**Fig 1:**
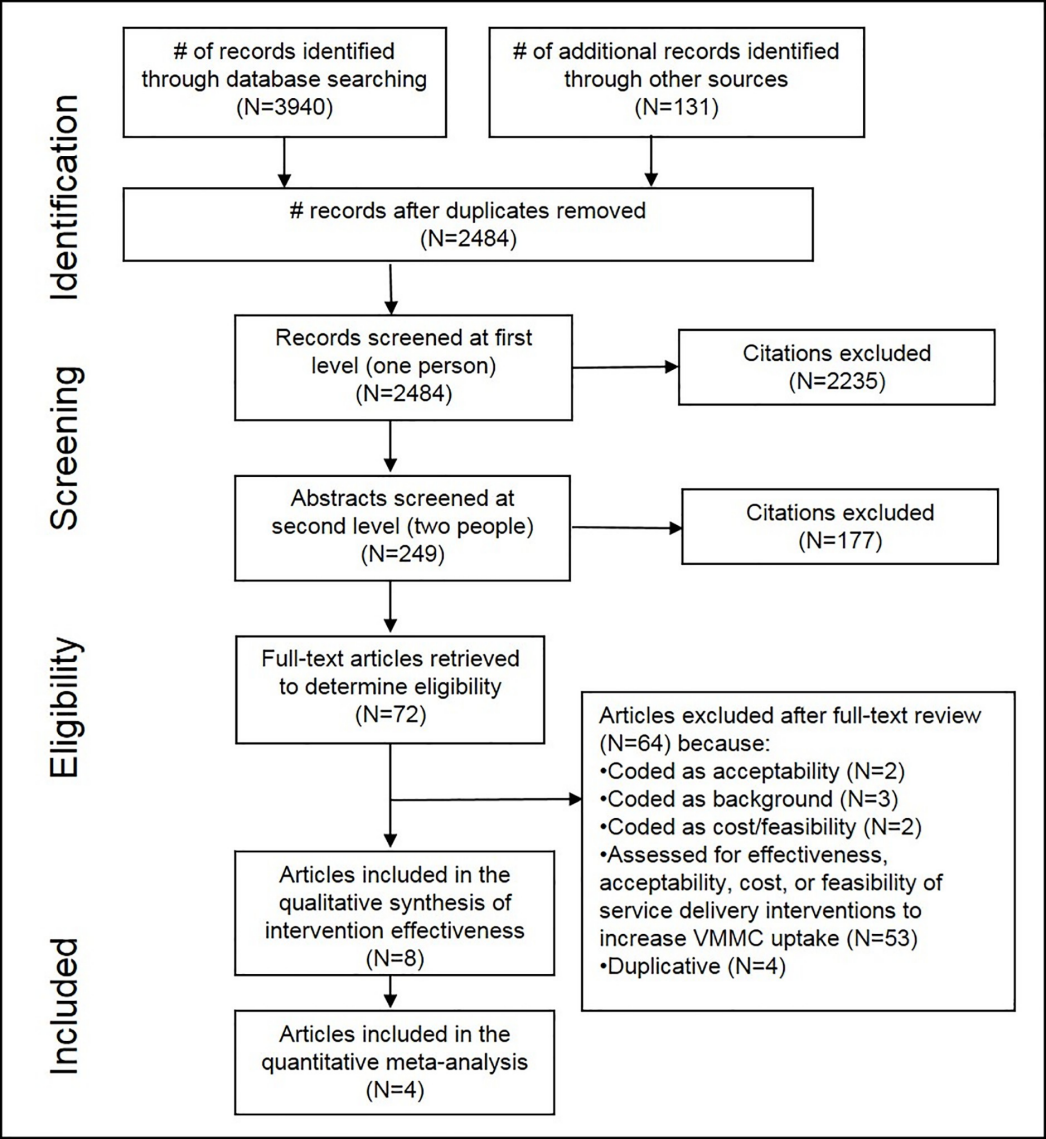
Flow of information through the different phases of a systematic review.

Electronic database searching retrieved 3,940 results, and an additional 131 were identified through searching conference databases, trial registries, contacting experts in the field, and secondary searching (Fig 1). After removing duplicates, there were 2,484 unique citations. After initial screening of titles and abstracts, 249 citations remained. After two independent reviewers screened these citations in duplicate, we selected 72 articles for full-text review. Of these, we included eight studies in the effectiveness review [24–31].

#### Description of included studies

Five RCTs [24, 27–30] and three non-randomized controlled trials [25, 26, 31] examined the effectiveness of various economic compensation strategies to increase uptake of VMMC. Two studies took place in Kenya [27, 28], and one each in Malawi [29], South Africa [30], Tanzania [24], Uganda [26], Zambia [31], and Zimbabwe [25]. Study populations included adult men 18 years or older [24, 26–31], except for one study targeting adolescent male secondary school students [25]. Four studies took place in rural communities [24, 27, 28, 31] and four in urban settings [25, 26, 29, 30]. Intervention approaches to increase VMMC uptake included lotteries [24, 28], food/transport vouchers [26–28], lowering the end-user’s price of VMMC services [29], conditional cash transfers [30], cash payment for VMMC referral [31], and non-monetary gifts [25]. Table 1 presents a summary description of included studies.

**Table 1:** Description of included studies–effectiveness review.

Study	Location, Population, Study design	Economic compensation intervention	Other intervention components	Comparison
**Lottery**				
Bazant et al., 2016 [24]	Tanzania (rural) Men age 20+ (n = 1186) RCT (cluster)	Demand creation lottery: smartphone raffle for VMMC clients (weekly) and health care providers and peer promoters (monthly)	Mass media campaigns and peer promoters	Mass media campaigns and peer promoters
Thirumurthy et al., 2016 [28]	Kenya (rural) Men age 21–39 (n = 903) RCT	Lottery-based rewards—Opportunity to participate in lottery with high-value prizes (1 in 20 would win a bicycle or smartphone (US$120), 2 in 20 a standard mobile phone or pair of shoes (US$45), 17 of 20 food voucher compensation (US$2.50))		Compensation of 50 Kenyan shillings (US$0.60) conditional on VMMC uptake
**Food and/or transport vouchers**				
Thirumurthy et al., 2014 [27]	Kenya (rural) Men age 25–49 (n = 1504) RCT	Food vouchers (valued 200 Kenya shillings (US$2.50), 700 shillings (US$8.75), or 1200 shillings (US$15.00)) as compensation for transportation costs / lost wages conditional on VMMC uptake. Note: These amounts approximated transportation costs from participants’ homes to a clinic or dispensary (≈US$2.50); transportation costs plus 1 to 2 days’ wages for most men based on the interquartile range of study participants’ daily wages (≈US$8.75); and transportation costs plus 2 to 3 days’ wages for most men based on the interquartile range of study participants’ daily wages (≈US$15)	Free VMMC services	Free VMMC services
Thirumurthy et al., 2016 [28] *(same as above)*	Kenya (rural) Men age 21–39 (n = 903) RCT	Fixed compensation—Food voucher (valued US$12.50) as compensation conditional on VMMC uptake		Compensation of 50 Kenyan shillings (US$0.60) conditional on VMMC uptake
Semeere et al., 2016 [26]	Uganda (urban) Male partners of pregnant women in 3^rd^ trimester attending antenatal care (n = 601) Non-randomized controlled trial	$8.50 transport voucher for male partner presenting for VMMC	Educating women about benefits of VMMC, procedure details, wound care, complications, how/when to get procedure, communication skill training, and information brochure	No intervention
**Lower price of VMMC services**				
Thornton et al., 2016 [29]	Malawi (urban) Men age 18–30 (n = 1649) RCT	Voucher for subsidized VMMC so that final price for male clients after using voucher was: free, 50 Malawi Kwacha (US$0.33), 100 Kwacha, 200 Kwacha, 500 Kwacha		Minimal subsidy: price after voucher 900 Kwacha *(cost with no voucher*: *950 Kwacha)*
**Cash**				
Wilson et al., 2016 [30]	South Africa (urban) Men age 18+ (n = 4000) RCT	Postcard offering a conditional cash transfer (South African Rand 100/US$10) for completing VMMC counseling session		Control 1 (true control): Postcard with basic information about VMMC Control 2: Postcard with a challenge message (“Are you tough enough?”) Control 3: Postcard with information on a possibly unknown benefit of VMMC (partner preference)
Zanolini et al., 2016 [31]	Zambia (rural) Men age 18+ (n = 699) Non-randomized controlled trial	Clients undergoing VMMC in intervention facilities could refer up to 5 uncircumcised men in their social network using referral vouchers and receive a monetary payment of 10 Zambian kwacha (US$2) for each referral		No intervention
**Non-monetary gift**				
Kaufman et al., 2016 [25]	Zimbabwe (urban) Adolescent male secondary school students age 14–20 (n = 565) Non-randomized controlled trial (nested within an RCT)	Nonmonetary incentive: free t-shirt or ticket to a soccer match	Make the Cut Plus (MTC+): Trained “coach” (circumcised man aged 18–30) facilitated interactive game (metaphor for HIV protection), personal story shared by coach, and group discussion; coach followed up with students and facilitates transport to VMMC clinic	MTC+ without nonmonetary incentives

RCT, randomized controlled trial; VMMC, voluntary medical male circumcision

#### Risk of bias

Risk of bias assessments of included studies are summarized in S1 and S2 Tables. The five RCTs included in this review had low risk of bias overall [24, 27–30]; additional risk of bias information for one RCT was obtained from a related publication [32]. Although the RCTs were not blinded, we judged them to have low risk of performance or detection bias as lack of blinding was unlikely to influence uptake outcomes. The three non-randomized controlled trials studies showed moderate risk of bias; while unrandomized, they all had comparison groups [25, 26, 31].

#### Uptake of VMMC: Overall analysis

**Fig 2:**
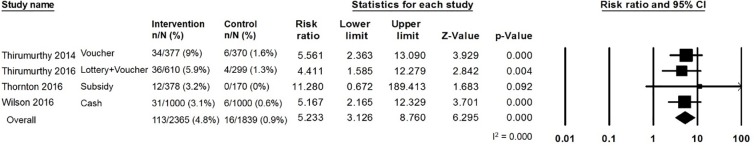
Meta-analysis results: Risk ratio of uptake of VMMC from RCTs, overall.

Combining effect size data from four RCTs of all types of economic compensation interventions, meta-analysis showed that economic compensation increased the uptake of VMMC compared with control groups that did not receive such interventions, or that received lesser forms of the interventions (RR: 5.23, 95% CI: 3.13 to 8.76, Q = 0.41, p = 0.94, I^2 =^ 0.00) (Fig 2) [27–30]. The meta-analysis used additional, unpublished data received from the authors of two studies; these new data are presented in Fig 2 [29,30]. One RCT presenting difference-in-differences was not combinable in meta-analysis; this study found a 47% increase in VMMC client attendance in the intervention group compared to an 8% increase in the comparison group [24]. However, across studies, the overall uptake of VMMC was low (range: 2.3–15.4% in intervention groups; 0–9.5% in comparison groups), and the absolute difference between groups was often relatively small.

We also examined the impact of different types of economic compensation interventions separately on VMMC uptake.

#### Uptake of VMMC: Lottery incentives

Two RCTs examined VMMC uptake comparing lottery incentives to control [24, 28]; however, these were not combinable in meta-analysis. One RCT presented a difference-in-differences analysis. In the intervention group, there was a 47% increase in VMMC client attendance (264 procedures 1 year before the study period versus 388 during the study period) compared to an 8% increase in the control group (257 before versus 278 during) [24]. A second RCT showed no statistically significant increase in VMMC uptake with lottery incentives alone (RR: 2.48, 95% CI: 0.79 to 7.81) [28].

#### Uptake of VMMC: Food and/or transport vouchers

Two RCTs provided evidence for the impact of food and/or transport vouchers [27, 28]. Meta-analysis showed an almost six-fold increase in likelihood of VMMC uptake associated with food and/or transport vouchers (RR: 5.85, 95% CI: 3.02 to 11.34, Q = 0.03, p = 0.85, I^2^ = 0.00). An additional non-randomized trial found that transport vouchers for male partners of pregnant women attending antenatal care in the third trimester had no statistically significant impact on VMMC uptake (RR: 1.69, 95% CI: 0.50 to 5.74) [26].

#### Uptake of VMMC: Subsidized VMMC services

One RCT examined the impact of lower-priced VMMC services (at a private clinic) compared to no subsidy for surgery costs [29]. This study showed a large but non-statistically significant increase in VMMC uptake, and there were serious concerns around imprecision (RR: 11.28, 95% CI: 0.68 to 189.42).

#### Uptake of VMMC: Conditional cash transfers

One RCT assessed the influence of conditional cash transfers for completing a VMMC counseling session. Authors of the RCT provided additional data stratified by study arm which was not available in the original article. The study showed that conditional cash transfers led to greater VMMC uptake (RR: 5.17, 95% CI: 2.17 to 12.33) [30]. A non-randomized trial found an interaction effect showing that small monetary payments for VMMC client referrals was associated with a non-statistically significant increase of 7.60 circumcisions per month (95% CI: -20.37 to 40.83) [31].

#### Uptake of VMMC: Non-monetary gifts

One cross-sectional study conducted among secondary school students ages 14–20 found no impact on VMMC uptake when offered a non-monetary gift (i.e. t-shirt or ticket to a soccer match) for VMMC completion (RR: 1.62, 95% CI: 0.88 to 2.98) [25].

#### Uptake of VMMC: Stratified analyses

No included studies specifically looked at high-risk men. In terms of age, all studies were among adult men except for one [25], which was also the only study to look at non-monetary gifts, making it difficult to discern the impact of interventions by age. Four studies took place in urban settings and four in rural; there were no clear trends in uptake of VMMC by location.

#### Other outcomes

No comparative data for economic compensation interventions were found for the other outcomes of interest: (2) uptake of HIV testing within VMMC services, (3) uptake of safer sex and risk reduction counseling within VMMC services, (4) changes in community expectations for economic compensation for other services, and (5) potential coercion (undue influence) on individuals or groups within the community.

#### Acceptability

We identified six studies that examined acceptability of economic compensation intervention: four qualitative studies and two quantitative surveys (Table 2) [24, 31, 33–36]. These studies suggested incentives are generally acceptable, valued for addressing key barriers, and motivating to men. However, some participants felt they were not sufficiently motivating or were unnecessary, and one study suggested they might raise community suspicions [24].

**Table 2:** Description of included studies–acceptability review.

Study	Location	Methods/Participants	Acceptability findings
Bazant et al., 2016 [24] (also in effectiveness review)	Tanzania	Focus group discussions 6 focus groups with 40 VMMC clients and 6 with 32 peer promoters	Peer promoters said the smartphone raffle succeeded in creating “buzz” for VMMC. However, several participants said the raffle raised community suspicions. Some men wondered why the phone was not the older model they knew. Some felt the smartphone was too expensive and out of touch. Others preferred an incentive that all clients could receive. Money was most frequently recommended incentive; suggested amounts ranged from TSh 1000 to TSh 20,000 (US $0.54–$10.81). Some said all VMMC clients should receive transportation reimbursement, transportation to the facility, food to take home, or farming commodities. Some clients and peer educators believed a free good-quality service was incentive enough.
DeCelles et al., 2016 [33] (related to Kaufman et al., 2016 [25] in effectiveness review)	Zimbabwe	Qualitative interviews and focus groups 17 interviews and 2 focus groups with coaches and 29 interviews with circumcised (n = 13) and uncircumcised boys (n = 16) ages 14–19	There were mixed reactions to the incentives. Some participants felt that incentives increased their motivation to go for VMMC. Others felt that the “Coach’s Story,” a story told by a circumcised facilitator about his experience receiving VMMC, was a more important motivating factor. Overall acceptability was high for both the t-shirt and tickets as incentives. Some preferred the tickets because of their strong interest in soccer. Others preferred the t-shirt, which coaches believed stemmed from their desire to wear the same shirt as their coaches.
Evens et al., 2014 [34]	Kenya	Qualitative interviews and focus groups Interviews with 8 circumcised and 14 uncircumcised men, 20 female partners, 12 health providers, 12 community leaders and 12 employers; 8 focus groups.	The most highly prioritized intervention to address financial concerns among men was the provision of money to compensate for lost wages and/or provide for family needs such as food, rent or children’s school fees. While most providers and community leaders supported this intervention, a number felt that providing cash was neither necessary nor feasible. Community leaders felt men do not need financial support after circumcision, either because the actual need for assistance was low, because men would not want to take money from others or because they would not want their VMMC decisions to be public knowledge. The provision of food or food vouchers to men following VMMC was also discussed.
Evens et al., 2016 [35] (related to Thirumurthy et al., 2016 [28] in effectiveness review)	Kenya	Qualitative interviews 45 circumcised and uncircumcised men and 19 female partners	Compensation promoted VMMC uptake by addressing lost wages. Participants who did not get circumcised perceived the amounts to be insufficient for offsetting their costs, or reported nonfinancial barriers such as fear of pain. Participants did not feel compelled to get circumcised for financial gain. Female partners felt the intervention motivated their partners to get circumcised.
Marshall et al., 2017 [36]	South Africa	Quantitative survey 142 men undergoing VMMC	Financial compensation was reported as important or very important by 37.4% (53/142); almost 40% (56/142) reported that they would not have undergone circumcision without this compensation element (p = 0.023).
Zanolini et al., 2016 [31] (also in effectiveness review)	Zambia	Quantitative survey 289 men ages 18+ undergoing VMMC	65% reported that the referral incentive motivated them to refer friends for VMMC “a lot,” and 35% reported that it motivated them “only somewhat” (29%) or “not at all” (6%). 18% reported that the incentive did not motivate them enough because the amount was too low and another 12% because they were reluctant to discuss VMMC with their friends. Men who attempted referrals and men who did not were no different in terms of age, education, transportation cost, or knowledge of circumcision status of friends.

### Values and preferences

No studies were identified for the review on end-users’ and providers' values and preferences for the outcomes of interest.

### Costs

One study (also included in the effectiveness review) presented cost data from Soweto, South Africa [30]. Costs (excluding clinical costs) were calculated for economic incentives (US$10), postcards (US$2), and refreshments (US$1). The program cost US$91 per additional circumcision. The calculated cost per HIV infection averted ranged from US$450 to US$1350.

### Feasibility

We identified no studies that specifically looked at additional considerations around feasibility of economic incentives for VMMC.

## Discussion

Our review found evidence from five RCTs that economic compensation interventions were generally associated with improved uptake of VMMC. Evidence from three additional non-randomized controlled trials showed mixed impact on VMMC uptake. In cases where effects were not statistically significant, they generally showed trends in a positive direction. This overall positive effect of economic compensation interventions on VMMC uptake is encouraging and in line with findings of previous reviews [9–12]. However, while the relative effects were often appreciable, the overall uptake of VMMC in these studies was low, and the absolute differences between groups were small. As Carrasco et al. [9] have noted, economic compensation may have provided a final nudge toward VMMC uptake for men who had already been considering undergoing circumcision, but the absolute effect may be limited. Further, the included studies had relatively short follow-up times appropriate to the study context; in the longer programmatic timeframe of VMMC scale-up, even small absolute effects may add up over time.

While our positive findings are encouraging, their generalizability to current VMMC target groups may be limited for several reasons. First, the included studies took place against a backdrop of rapid VMMC scale-up [4]. It is plausible that uncircumcised men in these countries now bear little resemblance to the study populations, or those with economic constraints may represent a larger proportion of men who have not yet sought circumcision. Second, the included studies were predominantly conducted in countries where scale-up of VMMC has been relatively successful [4]. Our findings may be less applicable to countries that have not had as much success, although these are the very countries which might be most motivated to consider incentives. In the changed context of VMMC scale-up and across different countries, it is unclear to what extent our review findings would apply to new target populations.

Our review combined a wide variety of economic compensation interventions, which likely have different mechanisms of effect. Our findings suggest, for example, that food/transport vouchers and conditional cash transfers may be more effective than lotteries, subsidized VMMC, and receiving a free t-shirt/soccer ticket. Both food/transport vouchers and conditional cash transfers provide guaranteed compensation, compared with lotteries or a lowered price of VMMC, and may be more highly valued than receiving non-monetary gifts. In a recent review, Galárraga and Sosa-Rubí have noted that different economic incentives may influence behaviour through different pathways, including by inducing price or income effects, or by affecting psychological heuristics and biases such as discounting or present bias, habit formation, salience, or cognitive errors [12]. Further research could include examining these mechanisms of effect in addition to the overall effectiveness of economic compensation interventions.

Acceptability of economic compensation interventions was generally positive but only assessed in a few studies. There were a few potential concerns about either ineffectiveness or raising community suspicions. The single cost study found a cost per HIV infection averted of US$450-$1350, well in line with what is considered cost-effective in HIV prevention and cost-saving compared with HIV treatment costs [37]. These factors, as a complement to effectiveness, are critical to understanding the overall value of this intervention approach, but are likely to vary across settings. Local formative work and community engagement will be important before implementing any economic compensation interventions. Intervention tailoring and community engagement would need to consider issues surrounding equity and ethics of incentives and compensation, the amount and type of incentives or compensation, and the practicalities of delivering these interventions.

We identified no effectiveness data on several of our outcomes of interest: uptake of HIV testing in VMMC services, safer sex/risk reduction counseling in VMMC services, changes in community expectations for economic compensation for other services, and potential coercion. The first two of these outcomes–uptake of HIV testing and safer sex/risk reduction counseling in VMMC services–are part of the standard package of VMMC services, and thus may not have been measured separately in studies. The remaining two outcomes–changes in community expectations for compensation and potential coercion–may be less likely to be measured as outcomes in evaluation studies. We also identified no studies for the values and preferences or feasibility reviews; however, our focus on peer-reviewed articles and conference abstracts may have precluded us from finding insights from unpublished program reports or other grey literature documents. We encourage future studies to consider examining these gaps in the literature.

VMMC programs for HIV prevention have had enormous success in sub-Saharan Africa. Generally, VMMC is already provided free of charge in priority countries, and governments may be hesitant to further increase HIV exceptionalism by providing additional economic compensation for VMMC. However, our review suggests that carefully selected economic interventions may be a useful targeted strategy to enhance VMMC coverage to achieve near-term targets in priority countries. Findings from this review directly informed forthcoming WHO guidelines on VMMC, which describe how such interventions may be considered with appropriate tailoring to local settings and adequate community engagement. They provide a guide for policymakers considering economic incentives for VMMC in the context of overall programming and goals.

## Supporting information

S1 Fig PRISMA checklist [18]. (DOC)

S1 Table Cochrane risk of bias tool for randomized controlled trials [21]. (DOCX)

S2 Table Evidence Project risk of bias tool for non-RCTs [22]. (DOCX)
