# Supplementary material for: Economic compensation interventions to increase uptake of voluntary medical male circumcision for HIV prevention: A systematic review and meta-analysis
Source: PLoS One. 2020 Jan 15;15(1):e0227623. doi: 10.1371/journal.pone.0227623 (PMC6961886; doi:10.1371/journal.pone.0227623)
Supplement: S1 Table — (DOCX) [file pone.0227623.s002.docx]

**S1 Table. Cochrane risk of bias tool for randomized controlled trials [21].**

| Study | Type of bias | Judgment | Support for Judgment |
| --- | --- | --- | --- |
| Bazant et al., 2016 [24]  Tanzania | Random sequence generation (selection bias) | Low risk | "One facility in each pair was randomly allocated in a coin toss to intervention or control." |
|  | Allocation concealment (selection bias) | Unclear risk | "Facilities were matched on region, patient volume, and facility type. [There were two exceptions. A hospital was matched to a large health center in Iringa. Also, a health center in Iringa was matched to a health center in Njombe.] … with the [random] selection made by government stakeholders." |
|  | Blinding of participants and personnel (performance bias) | Low risk | Blinding of participants and personnel not possible due to study design, but outcome (VMMC uptake) not likely to be influenced by lack of blinding. |
|  | Blinding of outcome assessment (detection bias) | Low risk | Blinding of outcome assessment not possible due to study design, but outcome (VMMC uptake) not likely to be influenced by lack of blinding. |
|  | Incomplete outcome data addressed (attrition bias) | Low risk | Reasons for missing outcome data unlikely to be related to true outcome; censoring unlikely to be introducing bias. |
|  | Selective reporting (reporting bias) | Low risk | The study protocol is not available but it is clear that the published reports include all expected outcomes, including the primary outcome that was pre-specified: “number of VMMCs among men aged 20 and older.” |
| Thirumurthy et al., 2014 [27]  Kenya | Random sequence generation (selection bias) | Low risk | "Seven of 78 sublocations in the study area were first randomly selected using selection probabilities proportional to each sublocation's population. [… household listing, names of all male household members, random sample of men selected for home visits…] Randomization was performed using computer-generated scratch cards and balanced block randomization (block size, 100)." |
|  | Allocation concealment (selection bias) | Low risk | "Scratch cards [randomized 1:1:1:1 to one of the four study groups] were offered to participants sequentially and revealed the study group assignment to the participant and research assistant at the same time." |
|  | Blinding of participants and personnel (performance bias) | Low risk | Blinding of participants and personnel not possible due to study design, but outcome (VMMC uptake) not likely to be influenced by lack of blinding. |
|  | Blinding of outcome assessment (detection bias) | Low risk | Blinding of outcome assessment not possible due to study design, but outcome (VMMC uptake) not likely to be influenced by lack of blinding. |
|  | Incomplete outcome data addressed (attrition bias) | Low risk | Reasons for missing outcome data unlikely to be related to true outcome; censoring unlikely to be introducing bias. |
|  | Selective reporting (reporting bias) | Low risk | All outcomes from study protocol are reported (primary outcome: "uptake of male circumcision within 2 months after men were given the opportunity to receive a food voucher"). |
| Thirumurthy et al., 2016 [28]  Kenya | Random sequence generation (selection bias) | Low risk | "Randomization was reformed using computer-generated scratch cards and balanced block randomization." |
|  | Allocation concealment (selection bias) | Low risk | "Scratch cards [randomized in a 1:1:1 ratio to three study groups] were offered to participants sequentially and revealed the study group assignment to the participant and research assistant simultaneously." |
|  | Blinding of participants and personnel (performance bias) | Low risk | Blinding of participants and personnel not possible due to study design, but outcome (VMMC uptake) not likely to be influenced by lack of blinding. |
|  | Blinding of outcome assessment (detection bias) | Low risk | Blinding of outcome assessment not possible due to study design, but outcome (VMMC uptake) not likely to be influenced by lack of blinding. |
|  | Incomplete outcome data addressed (attrition bias) | Low risk | Reasons for missing outcome data unlikely to be related to true outcome; censoring unlikely to be introducing bias. |
|  | Selective reporting (reporting bias) | Low risk | The study protocol is not available but it is clear that the published reports include all expected outcomes, including the primary outcome that was pre-specified: “VMMC uptake within 3 months of enrollment.” |
| Thornton et al., 2016 [29]  Malawi | Random sequence generation (selection bias) | Unclear risk | "Vouchers were randomized at the individual level." "Questionnaires [and vouchers] were given to enumerators in random order, shuffled by the authors." More detailed methods in related article for same study [32]. |
|  | Allocation concealment (selection bias) | Low risk | "Vouchers were placed in sealed envelopes and stapled to the back of the questionnaire." More detailed methods in related article for same study [32]. |
|  | Blinding of participants and personnel (performance bias) | Low risk | Blinding of participants and personnel not possible due to study design, but outcome (VMMC uptake) not likely to be influenced by lack of blinding. |
|  | Blinding of outcome assessment (detection bias) | Low risk | Blinding of outcome assessment not possible due to study design, but outcome (VMMC uptake) not likely to be influenced by lack of blinding. |
|  | Incomplete outcome data addressed (attrition bias) | Low risk | Reasons for missing outcome data unlikely to be related to true outcome; censoring unlikely to be introducing bias. |
|  | Selective reporting (reporting bias) | Low risk | The study protocol is not available but it is clear that the published reports include all expected outcomes, including the primary outcome that was pre-specified: “VMMC uptake within 3 months of enrollment.” |
| Wilson et al., 2016 [30]  South Africa | Random sequence generation (selection bias) | Low risk | "Outreach workers… [followed] a random walk method. In this random walk, outreach workers began distribution at prespecified locations, leaving the next postcard at every fifth house with an adult present, using coin-flips to determine the path at each intersection. The prespecified order randomized each postcard type and stratified on timing and location of distribution." |
|  | Allocation concealment (selection bias) | Low risk | "Outreach workers distributed postcards individually sealed in envelopes in a blinded, prespecified order to households" |
|  | Blinding of participants and personnel (performance bias) | Low risk | Blinding of participants and personnel not possible due to study design, but outcome (VMMC uptake) not likely to be influenced by lack of blinding. |
|  | Blinding of outcome assessment (detection bias) | Low risk | Blinding of outcome assessment not possible due to study design, but outcome (VMMC uptake) not likely to be influenced by lack of blinding. |
|  | Incomplete outcome data addressed (attrition bias) | Low risk | Reasons for missing outcome data unlikely to be related to true outcome; censoring unlikely to be introducing bias. |
|  | Selective reporting (reporting bias) | Low risk | The study protocol is not available but it is clear that the published reports include all expected outcomes, including the primary outcomes that were pre-specified: "uptake of the counseling session and of the procedures, as well as the initial possible step of calling the VMMC hotline.” |
