# Supplementary material for: Economic compensation interventions to increase uptake of voluntary medical male circumcision for HIV prevention: A systematic review and meta-analysis
Source: PLoS One. 2020 Jan 15;15(1):e0227623. doi: 10.1371/journal.pone.0227623 (PMC6961886; doi:10.1371/journal.pone.0227623)
Supplement: S2 Table — (DOCX) [file pone.0227623.s003.docx]

**S2 Table. Evidence Project risk of bias tool for non-RCTs [22].**

| Study | Study design includes | | | Participant representativeness | | | | Comparison groups equivalent at baseline on | |
| --- | --- | --- | --- | --- | --- | --- | --- | --- | --- |
|  | **Cohort** | **Control or comparison group** | **Pre/post intervention data** | **Participants randomly assigned to intervention** | **Participants randomly selected for assessment** | **Control for potential confounders** | **Follow-up rate ≥ 75%** | **Socio-demographics** | **Outcome measures** |
| Kaufman et al., 2016 [25]  Zimbabwe | Yes | Yes | Yes | No | Yes | No* | No | NR | NR |
| Semeere et al., 2014 [26]  Uganda | No | Yes | Yes | No | No | No | NA | Yes | NR |
| Zanolini et al., 2013 [31]  Zambia | No | Yes | Yes | No | No | No | NA | Yes | NA |

NA: not applicable, NR: not reported

*Regression was adjusted for school-level clustering and school type
